# Genomic Analysis of Group B *Streptococcus* Carriage Isolates From Botswana Reveals Distinct Local Epidemiology and Identifies Novel Strains

**DOI:** 10.1093/ofid/ofad496

**Published:** 2023-10-03

**Authors:** Karen L Hanze Villavicencio, Megan J Job, Anne Claire Burghard, Allison Taffet, Francis M Banda, Moses Vurayai, Margaret Mokomane, Tonya Arscott-Mills, Tiny Mazhani, Seeletso Nchingane, Brady Thomas, Andrew P Steenhoff, Adam J Ratner

**Affiliations:** Division of Infectious Diseases, Children's Hospital of Philadelphia, Philadelphia, Pennsylvania, USA; Department of Pediatrics, University of Pennsylvania Perelman School of Medicine, Philadelphia, Pennsylvania, USA; Department of Pediatrics, NewYork University Grossman School of Medicine, New York, New York, USA; Department of Pediatrics, NewYork University Grossman School of Medicine, New York, New York, USA; Renaissance School of Medicine, Stony Brook University, Stony Brook, NewYork, USA; Department of Pediatrics, NewYork University Grossman School of Medicine, New York, New York, USA; Department of Pediatrics & Adolescent Health, Faculty of Medicine, University of Botswana, Gaborone, Botswana; School of Allied Health Professionals, Faculty of Health Sciences, University of Botswana, Gaborone, Botswana; School of Allied Health Professionals, Faculty of Health Sciences, University of Botswana, Gaborone, Botswana; Department of Pediatrics & Adolescent Health, Faculty of Medicine, University of Botswana, Gaborone, Botswana; Botswana-UPenn Partnership, Faculty of Health Sciences, University of Botswana, Gaborone, Botswana; Global Health Center, Children's Hospital of Philadelphia, Philadelphia, Pennsylvania, USA; Department of Pediatrics & Adolescent Health, Faculty of Medicine, University of Botswana, Gaborone, Botswana; Department of Pediatrics, Princess Marina Hospital, Gaborone, Botswana; Department of Pediatrics, Stead Family Children's Hospital, University of Iowa, Iowa City, Iowa, USA; Division of Infectious Diseases, Children's Hospital of Philadelphia, Philadelphia, Pennsylvania, USA; Department of Pediatrics, University of Pennsylvania Perelman School of Medicine, Philadelphia, Pennsylvania, USA; Department of Pediatrics & Adolescent Health, Faculty of Medicine, University of Botswana, Gaborone, Botswana; Botswana-UPenn Partnership, Faculty of Health Sciences, University of Botswana, Gaborone, Botswana; Global Health Center, Children's Hospital of Philadelphia, Philadelphia, Pennsylvania, USA; Department of Pediatrics, NewYork University Grossman School of Medicine, New York, New York, USA; Department of Microbiology, NewYork University Grossman School of Medicine, New York, New York, USA

## Abstract

In pregnant people colonized with group B *Streptococcus* (GBS) in Botswana, we report the presence/expansion of sequence types 223 and 109, a low rate of erythromycin resistance, and 3 novel sequence types. These data highlight the importance of local epidemiologic studies of GBS, a significant source of neonatal disease.

Group B *Streptococcus* (GBS) is a major cause of neonatal sepsis and meningitis and a leading cause of death in infants globally [1, 2]. Rectovaginal colonization in late pregnancy can lead to transmission to newborns and is the major risk factor for early-onset GBS disease. Sub-Saharan African countries carry much of the burden of invasive GBS disease [1]. In the United States and some other high-income countries, pregnant people are routinely screened for rectovaginal colonization in late gestation, and targeted intrapartum antibiotic prophylaxis (IAP) is highly effective for prevention of early-onset GBS disease [3]. However, IAP has no significant effect in preventing late-onset disease and has not been implemented widely in low- and middle-income countries. Furthermore, rates of GBS resistance to second-line drugs such as erythromycin and clindamycin have increased, and strains with reduced β-lactam susceptibility (RBLS), though rare, have become more common [4, 5].

Vaccines targeting GBS capsular polysaccharide are in development and offer a promising alternative to IAP. However, there are 10 known GBS serotypes (Ia, Ib, and II–IX), and current protein–capsular polysaccharide conjugate vaccine candidates cover only a subset of these [6]. In addition, immunogenicity of multivalent GBS vaccines may vary by serotype [7]. There is significant geographic variability in GBS serotype distribution, strain backgrounds as determined by multilocus sequence typing (MLST), clonal complex (CC) assignment, and antibiotic resistance [1, 8]. Therefore, local epidemiologic studies are important for understanding the potential utility of candidate vaccines. Our group previously reported GBS rectovaginal colonization rate and serotype distribution in a cohort of pregnant people in Botswana, demonstrating local predominance of serotype V (>45% of isolates compared with approximately 20% worldwide) [9, 10]. Here, we further characterize GBS isolates from that cohort by whole-genome sequencing (WGS) to determine MLST/CC, presence of specific bacterial virulence determinants, and antimicrobial susceptibility.

## METHODS

*Streptococcus agalactiae* isolates were grown on selective agar (CHROMagar StrepB) at 37°C for 18–24 hours. Purple colonies, indicating GBS growth, were used to inoculate overnight cultures in 5 ml of tryptic soy broth. Bacteria were pelleted, resuspended in 300 mL of Tissue & Cell Lysis Solution (LGC Biosearch), and underwent bead beating with 0.1-mm zirconia/silica beads for 6 minutes. Genomic DNA was extracted using the MagMAX Viral/Pathogen Ultra Nucleic Acid Isolation Kit (Applied Biosystems) on a KingFisher Flex platform. Library preparation and WGS were performed on the Illumina NovaSeq 6000 platform (paired end; 150–base pair reads). Trimmomatic 0.36 software was used for adapter sequence removal and quality trimming, with a minimum read length of 120 base pairs.

We used SRST2 0.2.0 software for read mapping to assign genomic serotype using the GBS-SBG database [11], MLST/CC using *S agalactiae* allele sequences and MLST definition files from PubMLST [12], and antimicrobial resistance and protein profiles using databases from Metcalf et al [13]. We detected the presence of AlpST-1, a more distantly related member of the Alp protein family, by read-mapping to the AlpST-1 open reading frame from GBS SS1 [14]. Phenotypic determination of antimicrobial susceptibility was not performed. We used Mashtree 1.2.0 software to calculate a kmer distance tree and visualized it on the Microreact web server [15, 16]. Raw sequencing reads are available in the National Center for Biotechnology Information Sequence Read Archive (under BioProject identifier PRJNA986888). Isolate data are in the PubMLST database (PubMLST.org; accession nos. 25868–25908). This study of deidentified samples did not include factors necessitating patient consent.

## RESULTS

**Figure 1.**
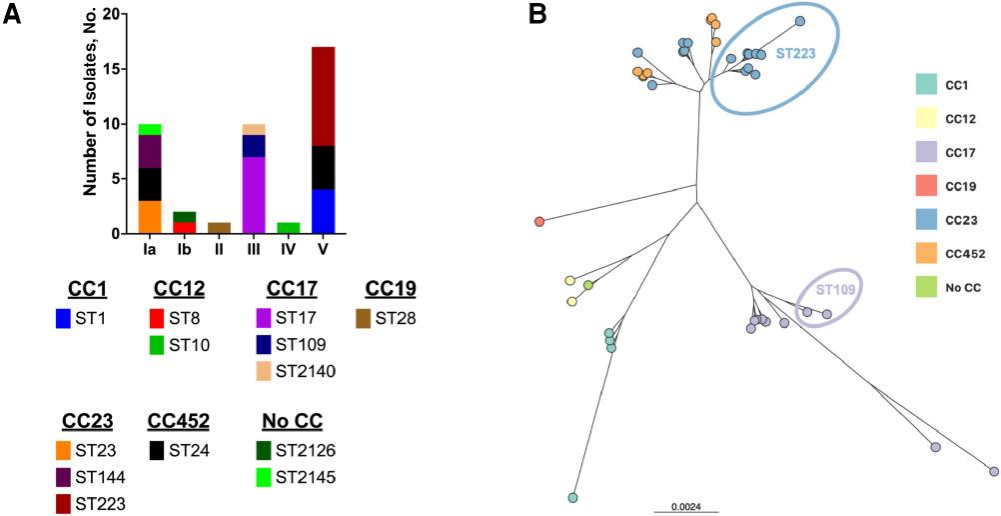
*A*, Serotype, sequence type (ST), and clonal complex (CC) of group B *Streptococcus* (GBS) isolates from pregnant people in Botswana. *B*, GBS whole-genome similarity was assessed using Mashtree 1.2.0 and visualized using the Microreact web server. Nodes are shaded by CC. Branch lengths and scale bar represent Mash distances. Specific ST groupings discussed in the text (ST109 and ST223) are indicated.

Of 53 GBS-positive samples from the original study, we were able to recover and generate high-quality genome sequences from 41 (77.4%). Serotype and MLST/CC data are presented in Figure 1*A*. Predicted serotypes were consistent with prior polymerase chain reaction–based serotyping [9]. We noted that sequence type [ST] 223 was the predominant ST [9 of 41 [22.0%]], followed by ST24 [7 of 41 [17.1%]] and ST17 [7 of 41 [17.1%]). A tree based on genome-wide genetic distances demonstrates a high degree of relatedness of the ST223 strains from this study (Figure 1*B*). The most common CCs were CC23 (15 of 41 [36.6%]), CC17 (10 of 41 [24.4%]), and CC452 (7 of 41 [17.1%]). A minority of isolates (3 of 41 [7.3%]) had MLST profiles not previously described. These were deposited in the PubMLST database and assigned STs 2126 (strain AR1534), 2140 (strain AR1537), and 2145 (strain AR1563). Complete isolate-level data appear in Table 1.

**Table 1.** Sequence Types, Clonal Complexes, Serotypes, Antibiotic Susceptibility Markers, and Surface Protein Genes of Group B *Streptococcus* Isolates as Determined by Whole-Genome Sequencing

Strain	ST	CC	Serotype	MLS Resistance	Tetracycline Resistance	PBP2x Mutations	Alp	Pilus	Srr1/2	HvgA
AR1515	24	CC452	V	…	*tetM*	T175A; I377V; V510I	Alpha	PI-2a	Srr1	…
AR1516	17	CC17	III	…	*tetM*	I377V; G627V	Rib	PI-1; PI-2b	Srr2	HvgA
AR1517	144	CC23	Ia	…	*tetM*	I377V; V510I	Rib	PI-1; PI-2a	Srr1	-
AR1518	109	CC17	III	…	*tetM*	I377V; G398A; G627V	Rib	PI-1; PI-2b	Srr2	HvgA
AR1519	223	CC23	V	…	*tetM*	I377V; G627V	Rib	PI-2a	Srr1	…
AR1521	1	CC1	V	*ermTR*	…	…	Alp2/3; AlpST-1	PI-1; PI-2a	Srr1	…
AR1522	223	CC23	V	…	*tetM*	I377V; G627V	Rib	PI-2a	Srr1	…
AR1523	223	CC23	V	…	*tetM*	I377V; G627V	Rib	PI-2a	Srr1	…
AR1525	223	CC23	V	…	*tetM*	I377V; G627V	Rib	PI-2a	Srr1	…
AR1526	223	CC23	V	…	*tetM*	I377V; G627V	Rib	PI-2a	Srr1	…
AR1527	17	CC17	III	…	*tetM*	I377V; G627V	Rib	PI-1; PI-2b	Srr2	HvgA
AR1528	28	CC19	II	…	*tetM*	…	Rib	PI-1; PI-2a	…	…
AR1529	223	CC23	V	…	*tetM*	I377V; G627V	Rib	PI-2a	Srr1	…
AR1531	10	CC12	IV	…	*tetM*	…	Alpha	PI-1; PI-2b	Srr2	…
AR1534	2126	No CC	Ib	…	*tetM*	…	Alpha	PI-1; PI-2a	Srr1	…
AR1535	8	CC12	Ib	…	*tetM*	…	Alpha	PI-1; PI-2a	Srr1	…
AR1537	2140	CC17	III	…	*tetM*	I377V; G627V	Rib	PI-1; PI-2b	Srr2	HvgA
AR1538	24	CC452	V	…	*tetM*	V510I	Alpha	PI-2a	Srr1	…
AR1539	1	CC1	V	*ermTR*	*tetM*	…	Alp2/3; AlpST-1	PI-1; PI-2a	Srr1	…
AR1540	24	CC452	V	…	*tetM*	I377V; V510I	Alpha	PI-2a	Srr1	…
AR1541	24	CC452	V	…	*tetM*	I377V; V510I	Alpha	PI-2a	Srr1	…
AR1542	223	CC23	V	…	*tetM*	I377V; G627V	Rib	PI-2a	Srr1	…
AR1543	24	CC452	Ia	…	*tetM*	I377V; V510I	Alpha	PI-2a	Srr1	…
AR1544	17	CC17	III	…	*tetM*	I377V; G627V	Rib	PI-1; PI-2b	Srr2	HvgA
AR1545	144	CC23	Ia	…	*tetM*	I377V; V510I	Rib	PI-2a	Srr1	…
AR1553	24	CC452	Ia	…	*tetM*	I377V; V510I	Alpha	PI-2a	Srr1	…
AR1555	223	CC23	V	…	…	I377V; G627V	Rib	PI-2a	Srr1	…
AR1558	23	CC23	Ia	…	*tetM*	I377V; V510I	Alp1	PI-2a	Srr1	…
AR1560	17	CC17	III	…	*tetM*	I377V; G627V	Rib	PI-1; PI-2b	Srr2	HvgA
AR1561	24	CC452	Ia	…	*tetM*	I377V; V510I	Alpha	PI-2a	Srr1	…
AR1563	2145	No CC	Ia	…	*tetM*	I377V; V510I	Alpha	PI-2a	Srr1	…
AR1723	23	CC23	Ia	…	*tetM*	I377V; V510I	Alp1	PI-2a	Srr1	…
AR1724	17	CC17	III	…	*tetM*	I377V; G627V	Rib	PI-1; PI-2b	Srr2	HvgA
AR1753	223	CC23	V	…	*tetM*	I377V; G627V	Rib	PI-2a	Srr1	…
AR1754	17	CC17	III	…	*tetM*	I377V; G627V	Rib	PI-1; PI-2b	Srr2	HvgA
AR1755	1	CC1	V	*ermTR*	*tetM*	…	Alp2/3; AlpST-1	PI-1; PI-2a	Srr1	…
AR1765	23	CC23	Ia	…	*tetM*	I377V; V510I	Alp1	PI-2a	Srr1	…
AR1771	144	CC23	Ia	…	*tetM*	I377V; V510I	Rib	PI-2a	Srr1	…
AR2184	17	CC17	III	…	*tetM*	I377V; G627V	Rib	PI-1; PI-2b	Srr2	HvgA
AR2189	1	CC1	V	*ermTR*	*tetM*	…	Alp2/3; AlpST-1	PI-1; PI-2a	Srr1	-
AR2287	109	CC17	III	…	*tetM*	I377V; G398A; G627V	Rib	PI-1; PI-2b	Srr2	HvgA

Abbreviations: Alp, Alpha-like protein; CC, clonal complex; HvgA, hypervirulent group B *Streptococcus* adhesin; MLS, macrolide-lincosamide-streptogramin; PBP, penicillin-binding protein; Srr, serine-rich repeat protein.; ST, sequence type.

Among the serotype III GBS isolates, 10 of 10 (100%) were members of the hypervirulent CC17 group. Most of these (7 of 10 [70%]) were ST17, but 2 were ST109, a GBS type previously described as a cause of invasive disease among children in Mozambique and Angola [17, 18]. ST109 has been reported to have an RBLS phenotype owing to the presence of a G398A mutation in penicillin-binding protein 2x [18]. Like the ST109 strains from the Mozambique study, both ST109 strains described here were found to have the G398A mutation. No other strains from the current study had G398A or other penicillin-binding protein 2x mutations known to be associated with a RBLS phenotype.

Nearly all Botswana GBS isolates (39/41 [95.1%]) harbored a *tetM* gene tetracycline resistance allele. Four isolates (4 of 41[9.8%]), all of which were serotype V/ST1, contained an *ermTR* gene that confers resistance to macrolides, lincosamides, and streptogramin B (MLS). No other MLS resistance determinants were identified, and no mutations in the 23S ribosomal subunit or the *rpo* genes were found. No changes in predicted quinolone resistance determinants were predicted by the analysis pipeline.

All strains contained ≥1 gene encoding a member of the alpha-like family of proteins (Rib, Alp1, Alp2/3, AlpST-1, or Alpha). AlpST-1 was detected in the 4 serotype V/ST1 strains that also encoded *ermTR* and *tetM* [14]. That combination of genotypic features is consistent with the ST1 subclade 2, previously reported by Cubria et al [19]. Consistent with prior data, CC17 strain genomes all had genes encoding Rib, pilus type 1, pilus type 2b, serine-rich repeat protein Srr2, and hypervirulent group B *Streptococcus* adhesin (HvgA). Non-CC17 strains were predicted to express Srr1, with 2 exceptions: strain AR1528 (II/CC19), which was not predicted to express either Srr1 or Srr2, and strain AR1531 (Ib/CC12), predicted to express Srr2.

## DISCUSSION

Africa has the highest estimated burden of invasive neonatal GBS cases, but epidemiologic data on circulating strains are still limited [1, 20]. GBS capsular serotype and MLST distributions differ among countries, with implications for potential efficacy of candidate vaccines. Here, we used bacterial WGS to determine serotypes, MLST, and the presence of genes encoding potential virulence factors and antimicrobial resistance genes in GBS strains from a previously described cohort of pregnant people in Botswana [9]. We noted an abundance (>20%) of serotype V, ST223 GBS strains. These findings differ from those of a prior study in coastal Kenya, in which <1% of GBS isolates from pregnant people were ST223 [21]. Similarly, in a GBS pangenome-wide association study, 6% of Malawian isolates were identified as ST223 [22]. In a sample of >6000 invasive GBS isolates from the United States, only 3 (<0.1%) were ST223 [23]. Notably, all of those US-based ST223 isolates were serotype Ia [23]. Local expansion of specific GBS types has been noted, including emergence of ST283 as a cause of invasive infections in humans and fish in southeast Asia and of ST459 in North America [24,25–26].Of the 6 major GBS CCs, CC17 is highly associated with sepsis and bacterial meningitis in infants in the first 90 days of life. Within CC17, ST109 strains are not commonly found but are of potential concern, given their association with both invasive disease and RBLS [17, 18]. Two ST109 isolates were identified in the current study, representing approximately 5% of GBS strains and raising the possibility that this GBS type may cause neonatal disease in Botswana and could be more widespread in the region than previously appreciated. We found that a low percentage of colonizing isolates (<10%) were predicted to be resistant to erythromycin, a rate lower than that reported in a recent meta-analysis from African nations (25.1%) [27]. We speculate that this finding may reflect a low rate of exposure to this class of antibiotics among the sampled population. In the United States, more than half of GBS strains are resistant to erythromycin [13, 23]. Fluoroquinolone resistance, which was not detected in this cohort, is rare in the United States (<2% of strains) [23]. The high rate of tetracycline resistance noted is consistent with findings that most human colonizing and invasive GBS isolates derive from tetracycline-resistant clones [28].

Taken together, these data carry messages of both hope and caution. All of the rectovaginal colonization isolates from this cohort of pregnant people in Botswana have capsular serotypes that are contained in the hexavalent vaccine candidate currently in clinical trials [7, 29]. In addition, rates of GBS resistance to erythromycin appear to be low in Botswana. However, local proliferation of the CC17/ST223 strain and detection of 2 ST109 isolates in this cohort underscore the need for expanded molecular epidemiologic studies of GBS in this region and for assessment of the role of emerging local strains in neonatal disease.
